# Impact of the Omicron variant on SARS-CoV-2 reinfections in France, March 2021 to February 2022

**DOI:** 10.2807/1560-7917.ES.2022.27.13.2200247

**Published:** 2022-03-31

**Authors:** Jonathan Bastard, Benjamin Taisne, Julie Figoni, Alexandra Mailles, Julien Durand, Myriam Fayad, Laurence Josset, Anna Maisa, Sylvie van der Werf, Isabelle Parent du Châtelet, Sibylle Bernard-Stoecklin

**Affiliations:** 1 Santé publique France, French national public health agency, Saint-Maurice, France; 2 Laboratoire de Virologie, Institut des Agents Infectieux, Laboratoire associé au Centre National de Référence des virus des infections respiratoires, Hospices Civils de Lyon, Lyon, France; 3 CIRI, Centre International de Recherche en Infectiologie, Team VirPath, Univ Lyon, Inserm, U1111, Université Claude Bernard Lyon 1, CNRS, UMR5308, ENS de Lyon, Lyon, France; 4 Molecular Genetics of RNA Viruses, Department of Virology, Institut Pasteur, CNRS UMR 3569, Université Paris Cité, Paris, France; 5 National Reference Center for Respiratory Viruses, Institut Pasteur, Paris, France

**Keywords:** SARS-CoV-2, reinfection, Omicron, COVID-19

## Abstract

Since the first reports in summer 2020, SARS-CoV-2 reinfections have raised concerns about the immunogenicity of the virus, which will affect SARS-CoV-2 epidemiology and possibly the burden of COVID-19 on our societies in the future. This study provides data on the frequency and characteristics of possible reinfections, using the French national COVID-19 testing database. The Omicron variant had a large impact on the frequency of possible reinfections in France, which represented 3.8% of all confirmed COVID-19 cases since December 2021.

Reinfections by severe acute respiratory syndrome coronavirus 2 (SARS-CoV-2) have been reported early after its emergence and rapid spread worldwide. The aim of this study was to describe the frequency and characteristics of possible reinfections with SARS-CoV-2 detected in France since March 2021.

## Temporal distribution of possible SARS-CoV-2 reinfections

We identified possible SARS-CoV-2 reinfections in the French national coronavirus disease (COVID-19) testing database. A case was defined as a person with at least two positive tests (by RT-qPCR, reverse transcription loop-mediated isothermal amplification (RT-LAMP) or antigen tests, excluding self-administered antigen tests, and irrespective of the variant involved) performed at least 60 days apart between 1 January 2021 and 20 February 2022. Because of changes in the patient anonymisation method in the database in early 2021, we could identify possible reinfections starting on 2 March 2021, but not those following a first episode or more in 2020.

**Figure 1:**
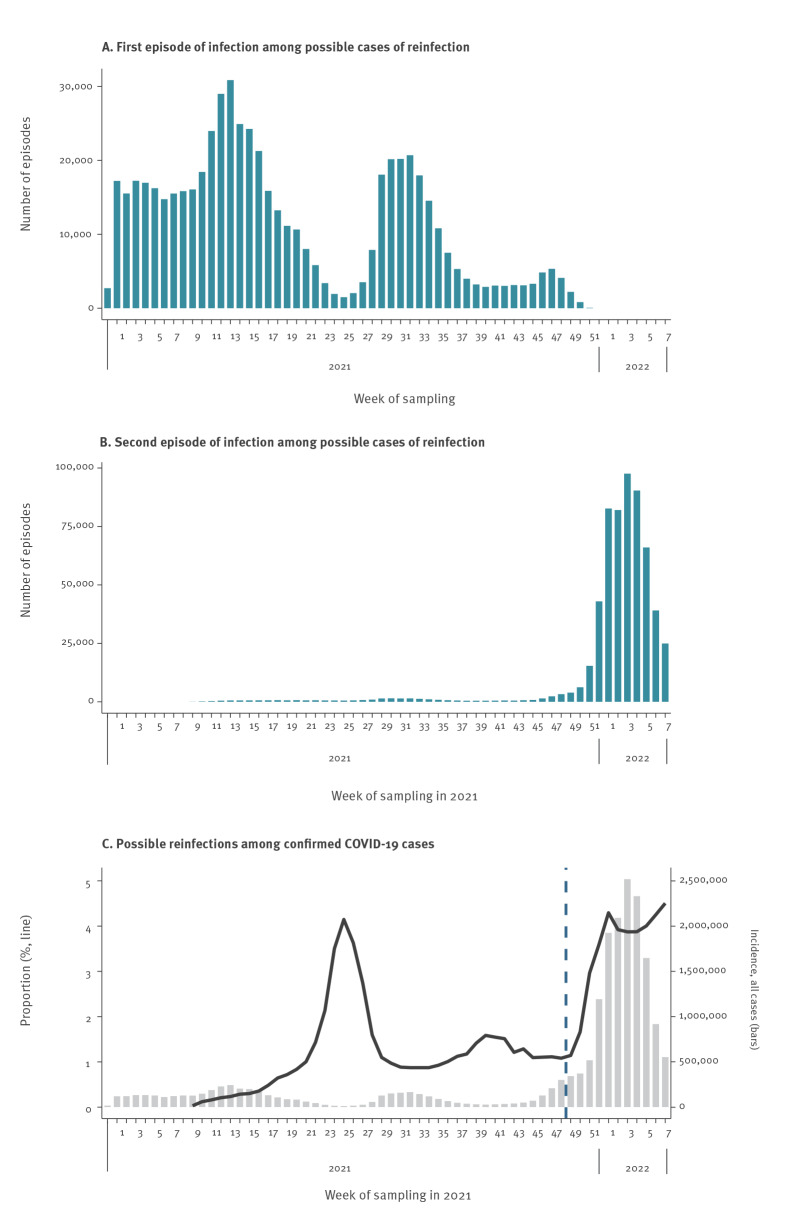
Distribution of the first and second episode of infection for possible cases of SARS-CoV-2 reinfection, and proportion of possible reinfections among all confirmed COVID-19 cases, by week of sampling, France, January 2021–February 2022 (n = 584,129)

COVID-19: coronavirus disease; SARS-CoV-2: severe acute respiratory syndrome coronavirus 2.

From 2 March 2021 to 20 February 2022, 589,702 possible reinfections were identified, including 584,129 people with two episodes (99.1%), 5,485 (0.9%) with three, 66 (< 0.1%) with four, and 22 people (< 0.1%) with between five and seven distinct episodes. We excluded all possible cases of SARS-CoV-2 reinfection with more than two distinct episodes, because (i) they were more likely to reflect persistent viral shedding in possibly immunosuppressed people than actual reinfections, and (ii) they represented less than 1% of the possible cases of reinfection. Among the 584,129 analysed cases, 390,835 (66.9%) had a first episode of infection between 1 January and 20 June 2021 (i.e. before the epidemic wave in summer 2021) (Figure 1A), and 551,182 (94.4%) had a second episode from 6 December 2021 onwards, i.e. after the introduction and rapid spread of the Omicron (Phylogenetic Assignment of Named Global Outbreak (Pango) lineage designation B.1.1.529) variant in France (Figure 1B).

Panels A and B: Because of the study period, we could only detect possible reinfections with a first episode of SARS-CoV-2 infection between weeks 53/2020 and 51/2021 and with a second episode from week 9/2021 onwards. Panel C: The vertical dashed line represents the time point where the Omicron variant exceeded 1% of all positive SARS-CoV-2 tests in France, between week 48 and 49 of 2021.

Possible cases of reinfection represented 3.1% of all confirmed COVID-19 cases detected between 2 March 2021 and 20 February 2022. This proportion varied over time, representing 0.8% in the period until week 48/2021 and 3.8% from the time of emergence of the Omicron variant (i.e. from week 49/2021 onwards, Figure 1C). In week 7/2022, possible cases of reinfection represented 4.5% of all confirmed cases.

## Characteristics of possible cases of SARS-CoV-2 reinfection

The mean time interval between two episodes of infection was 244 days, and the median was 267 days (interquartile range: 166–314). In a majority of cases (69.1%), this time period was 180 days or more (see Supplementary Table S1 for the numbers of possible reinfections by time interval).

The proportion of women, healthcare professionals and people aged 18 to 40 years was significantly higher among possible cases of reinfection compared with all confirmed COVID-19 cases (p < 0.001). In contrast, people younger than 18 years and older than 40 years were less frequent among possible cases of reinfection (p < 0.001) (Table). 

**Table:** Characteristics of possible cases of SARS-CoV-2 reinfection, compared with all confirmed COVID-19 cases, France, 2 March 2021–20 February 2022

	Possible cases of SARS-CoV-2 reinfection (n = 584,129)		All confirmed cases of COVID-19 (n = 18,661,139)		p value^a^
	n	%^b^	n	%^c^	
Female	324,253	55.6	9,862,846	53.4	< 0.001
Male	259,325	44.4	8,623,428	46.6	< 0.001
Healthcare professional	33,298	6.2	615,074	3.5	< 0.001
Symptoms reported	274,508^d^	51.3	8,831,873	51.3	> 0.05
Age group (years)					
< 18	137,915	23.6	5,052,111	27.1	< 0.001
18–40	298,230	51.1	7,127,055	38.2	< 0.001
41–60	116,799	20.0	4,495,255	24.1	< 0.001
61–80	22,420	3.8	1,584,451	8.5	< 0.001
> 80	8,265	1.4	397,037	2.1	< 0.001

COVID-19: coronavirus disease; SARS-CoV-2: severe acute respiratory syndrome coronavirus 2.

^a^ p value of a two-sample binomial proportion test.

^b^ Proportion among cases with available information. The percentage of missing values was 0.1% for the sex variable, 7.3% for healthcare professional status, 0.1% for the age variable and 8.3% for symptom reporting.

^c^ Proportion among cases with available information . The percentage of missing values was 1.0% for the sex variable, 6.8% for healthcare professional status, < 0.1% for the age variable and 7.8% for symptom reporting

^d^ Reporting symptoms at the time of sampling during the second episode of infection.

**Figure 2:**
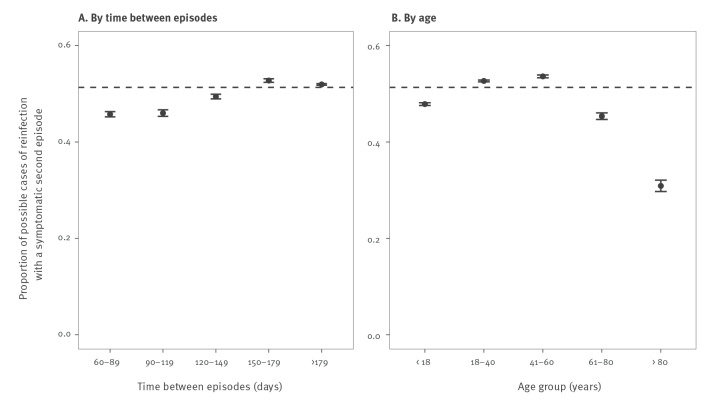
Proportion of possible cases of SARS-CoV-2 reinfection reporting symptoms during the second episode of infection, stratified by time between episodes and by age, France, March 2021–February 2022 (n = 535,539)

COVID-19: coronavirus disease; SARS-CoV-2: severe acute respiratory syndrome coronavirus 2.

The proportion of possible cases of reinfection reporting symptoms during their second episode was similar to that of all confirmed cases (p > 0.05) (Table). However, the shorter the time between two episodes, the smaller this proportion (Figure 2A). Symptoms were also less frequent during the second episode in people younger than 18 years or older than 60 years (Figure 2B).

Error bars represent the 95% binomial proportion confidence interval. The dashed horizontal line represents the proportion of symptomatic infections in all confirmed COVID-19 cases detected throughout the study period (see Table). Proportions are calculated among cases who provided information on the presence/absence of symptoms (values were missing in 8.3% of possible reinfections for the symptom reporting variable).

## Impact of Omicron on possible reinfections with SARS-CoV-2

In France, an RT-PCR screening strategy for SARS-CoV-2 mutations has been in place since 23 January 2021, with 26% of all confirmed COVID-19 cases screened for mutations carried by different SARS-CoV-2 variants of concern from June 2021 to February 2022. The targeted mutations changed over time in order to adjust to the variants circulating in France and abroad. We defined five categories of screening results for this analysis: suspicion of Alpha (B.1.1.7), Beta/Gamma (B.1.351/P.1), Delta (B.1.617.2), Omicron and 'Other', the latter including any other interpretable mutation screening result (see the Supplement for a summary of the RT-PCR screening strategy in France). In this section, we analysed only the possible reinfections that occurred in mainland France (i.e. 565,149 possible cases of reinfection, or 97% of the total).

An interpretable screening result was available for the first and/or the second episode in 53.5% (n = 302,604) of possible reinfection cases. First episodes were caused by several variants, while the large majority (86.1%) of the 117,601 possible cases of reinfection with an interpretable screening result on their second episode were most probably caused by Omicron.

**Figure 3:**
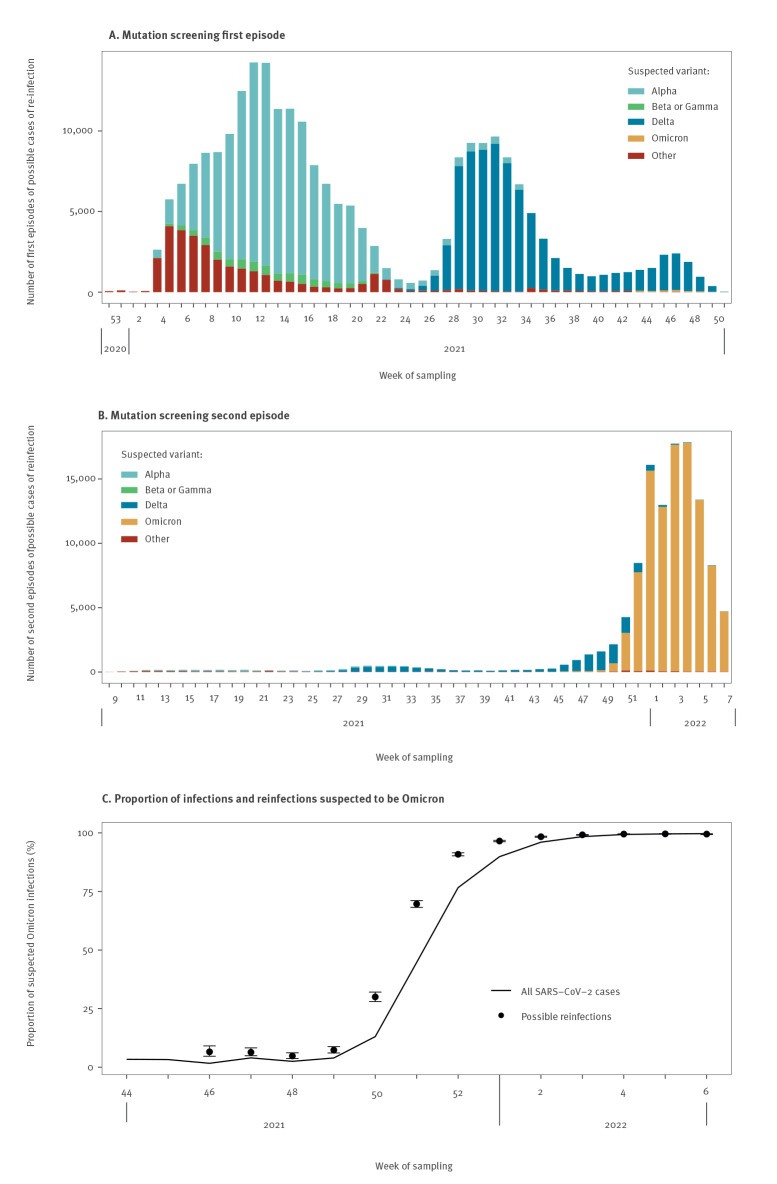
Suspected variants in possible cases of SARS-CoV-2 reinfection, by week of sampling, mainland France, January 2021–February 2022 (n = 302,604)

COVID-19: coronavirus disease; SARS-CoV-2: severe acute respiratory syndrome coronavirus 2.

Moreover, 10.6% (n = 59,760) of the 565,149 possible reinfections had an interpretable screening result for both episodes (Figure 3A and B). For 43.9% of them (n = 26,229), the results indicated a suspicion of infection with Alpha during the first episode and with Omicron during the second episode, and 31.5% (n = 18,842) had a suspicion of infection by Delta during the first episode and by Omicron during the second episode (see Figure 3A and B and the combinations of mutation screening results for both episodes in Supplementary Table S2). Possible reinfections with identical screening results for both episodes were rare, with a suspicion of Alpha, Delta and Omicron in both episodes in 613 (1.0%), 1,332 (2.2%) and 100 cases (0.2%), respectively.

SARS-CoV-2 mutation screening results were available in both first and second episodes for 59,760 possible cases of reinfection. Panel C displays weekly proportions of suspected Omicron infections among possible reinfections (dots and 95% binomial proportion confidence intervals) and among all confirmed SARS-CoV-2 infections (line).

Between week 46/2021 and week 3/2022, the proportion of infections suspected to be caused by the Omicron variant was systematically higher among possible reinfections than among all confirmed COVID-19 cases, even though the proportion of suspected Omicron infections rapidly increased in both categories (Figure 3C).

## Discussion

This study provides original data on possible reinfections by SARS-CoV-2 (i.e. people with two positive tests for SARS-CoV-2 at least 60 days apart) in France, generated from a nationwide, exhaustive database of COVID-19 testing results. While possible reinfections were a rare event (< 1% of all confirmed COVID-19 cases) during most of the study period, their frequency increased dramatically after the emergence and spread of the Omicron variant (December 2021 to February 2022), representing more than 4% of total confirmed COVID-19 cases in mid-February. This phenomenon could be due to the waning of post-infection and post-vaccination immunity in people infected and/or vaccinated earlier in the pandemic. Moreover, these reinfections most probably reflect the immune escape characteristics of Omicron. This is consistent with other reports published worldwide [1-7].

Interestingly, before the introduction and rapid diffusion of Omicron in France in early December 2021, the proportion of possible reinfections among confirmed COVID-19 cases fluctuated markedly, with high values observed during inter-epidemic periods, in June and September 2021. This could reflect the fact that during these periods of lower incidence, SARS-CoV-2 mostly circulated within populations with a higher exposure to the virus and who, possibly related to their profession, socioeconomic status or lower compliance to physical distancing, were more likely to have previously been infected and remained highly exposed during inter-epidemic periods.

The same hypothesis could explain the higher proportion of young adults and healthcare professionals among possible reinfections compared with all confirmed COVID-19 cases. On the contrary, the lower proportion of older adults could be due to a generally lower exposure to SARS-CoV-2 (e.g. better compliance to prevention measures such as physical distancing) and to a higher vaccination coverage (in Supplementary Table S3, we provide the vaccination coverage by age group during the study period), especially for those with a booster dose. This higher vaccination coverage could also explain why a lesser proportion of possible reinfections were symptomatic in this group compared with younger adults.

The majority of possible reinfections occurred more than 6 months after the first infection episode. However, ca 6% of possible reinfections had an interval of 60–89 days between episodes, indicating that a 90-day cut-off in the case definition of a SARS-CoV-2 reinfection – a cut-off adopted by several health agencies [8] – could lead to under-detection of reinfections. On the other hand, a fraction of the possible cases of reinfection – especially those occurring within a 90-day period – may be false-positives caused by prolonged viral detection, particularly in immunosuppressed [9,10] or severely ill people [11,12]. This could explain the detection of several cases with five or more positive tests at least 60 days apart. However, the proportion of false-positive cases of reinfection is likely to be negligible with a 60-day threshold, based on available literature [13,14].

The proportion of cases reporting symptoms during reinfection was similar to that of all confirmed COVID-19 cases. However, we observed a smaller proportion of symptomatic cases among possible reinfections with a shorter interval between infections. This suggests that the immune response may partly protect from a symptomatic reinfection shortly after a primary infection, but that such an effect tends to wane over time. Further work is needed to investigate this hypothesis.

This study has several limitations. Firstly, virological (e.g. viral load, sequencing data) and epidemiological (e.g. contact with a confirmed COVID-19 case before the second episode) information was not available, which could affect the specificity of our case definition for possible reinfection. Secondly, we did not have access to information on hospitalisation and vaccination status of possible cases of reinfection, which prevented a thorough characterisation of possible reinfections. In the future, matching the COVID-19 testing database with the national hospitalisation and vaccination databases would allow to further characterise the risk of SARS-CoV-2 reinfection. Finally, owing to technical specificities, we were not able to identify in this study possible reinfections with a first episode that occurred in 2020. However, the study period still allowed highlighting the impact of the Omicron variant on reinfections by comparing the periods before and after its emergence. Moreover, the available literature on the frequency of reinfections before the emergence of Omicron indicates that SARS-CoV-2 reinfections were rare events [15-19]. We therefore assume that the total number of undetected possible reinfections is small.

## Conclusion

The data presented here on possible SARS-CoV-2 reinfections provide valuable information on their characteristics and frequency over time. As it appears very unlikely that SARS-CoV-2 will be eradicated, monitoring the frequency of reinfections will be particularly useful in the future, especially in case of a new emerging variant. These data will be useful for decision-making regarding prevention and control strategies.

## Supplementary Data

172000 Supplement
