# Supplementary material for: Impact of the Omicron variant on SARS-CoV-2 reinfections in France, March 2021 to February 2022
Source: Euro Surveill. 2022 Mar 31;27(13):2200247. doi: 10.2807/1560-7917.ES.2022.27.13.2200247 (PMC8973013; doi:10.2807/1560-7917.ES.2022.27.13.2200247)
Supplement: Supplement [file 22-00247_BERNARD-STOECKLIN_Supplement.pdf]

Impact of Omicron on SARS-CoV-2 reinfections in France, March 2021 to February 2022

Supplementary materiel

This supplementary material is hosted by Eurosurveillance as supporting information alongside the article Impact of Omicron on SARS-CoV-2 reinfections in France, January 2021 to February 2022, on behalf of the authors, who remain responsible for the accuracy and appropriateness of the content. The same standards for ethics, copyright, attributions and permissions as for the article apply. Supplements are not edited by Eurosurveillance and the journal is not responsible for the maintenance of any links or email addresses provided therein

Supplementary method: RT-PCR screening strategy of SARS-CoV-2 mutations, France, 2021-2022

**Date of implementation**	**Targeted mutations**	**Type of results entered in the national testing database**	**Categories of screening results defined for the study on possible reinfections**
23/01/2021	N501Y **AND** DEL69-70 and/or A570D and/or K417N and/or E484K	**Nomenclature #1 :**<br>- Suspicion of Alpha<br>- Suspicion of Beta/Gamma<br>- Suspicion of wild type<br>- Other	From January to June 2021*, any result using nomenclature #1 :<br>Suspicion of Alpha<br>Suspicion of Beta/Gamma<br>Suspicion of wild type OR Other ► Other
31/05/2021	E484K **AND** E484Q **AND** L452R	**Nomenclature #2 :**<br>- Presence of the targeted mutations<br>- Absence of the targeted mutations<br>- Mutation not screened<br>- Uninterpretable result	From 31/05/2021 onwards :<br>Presence of L452R ► Suspicion of Delta<br>From 31/05/2021 until 29/08/2021 :<br>Absence of E484K **AND** of E484Q **AND** of L452R ► Suspicion of Alpha<br>From 01/11/2021 onwards :<br>Absence of E484K AND of L452R ► Suspicion of Omicron
29/11/2021	E484K **AND** E484Q **AND** L452R **AND** [DEL69/70 and/or K417N and/or N501Y]		From 29/11/2021 until 19/12/2021* :<br>Presence of at least one of the 3 mutations DEL69/70, K417N and N501Y ► Suspicion of Omicron
20/12/2021	E484K **AND** L452R **AND** [DEL69/70 and/or K417N and/or S371L-S373P and/or Q493R]		From 20/12/2021 onwards :<br>Presence of at least one of the 4 mutations DEL69/70, K417N, S371L-S373P and Q493R ► Suspicion of Omicron

Supplementary table 1: Distribution of possible cases of SARS-CoV-2 reinfection depending on the time interval between both episodes

Time interval between episodes	N	%
60-89 days	36,508	6.2
90-119 days	23,109	4.0
120-179 days	121,028	20.7
180-364 days	365,462	62.6
≥365 days	38,022	6.5

Supplementary table 2: Mutation screening results when available for both episodes among possible cases of SARS-CoV-2 reinfection (total: n=59,760)

		Suspected variant during the second episode of infection									
		Alpha		Beta / Gamma		Delta		Omicron		Other	
		N	%	N	%	N	%	N	%	N	%
Suspected variant during the first episode of infection	Alpha	613	1.0	3	0.0	2,318	3.9	26,229	43.9	354	0.6
	Beta/ Gamma	14	0.0	9	0.0	170	0.3	1,695	2.8	28	0.0
	Delta	0	0.0	0	0	1,332	2.2	18,842	31.5	161	0.3
	Omicron	0	0.0	0	0	0	0	100	0.2	0	0
	Other	102	0.2	3	0	816	1.4	6,811	11.4	160	0.3

Interpretable results for both COVID-19 episodes available for 59,760 possible cases of SARS-CoV-2 reinfection

Supplementary table 3: Vaccination coverage (primary vaccination and booster dose) among age categories, France, December 5th 2021 and February 20th 2022

Age categories	Population size	December 5th 2021				February 20th 2022			
		Primary vaccination		Booster dose		Primary vaccination		Booster dose	
		N	%	N	%	N	%	N	%
**< 18 years**	14,435,760	3,819,711	26.5	9,732	0.1	4,268,327	29.6	674,969	4.7
**18-39 years**	17,436,092	15,274,140	87.6	772,896	4.4	16,140,312	92.6	10,410,178	59.7
**40-59 years**	17,385,561	15,628,473	89.9	2,050,054	11.8	16,105,174	92.6	12,667,730	72.9
**60-79 years**	13,699,722	12,796,507	93.4	5,414,868	39.5	13,014,982	95.0	11,583,292	84.6
**> 79 years**	4,157,861	3,555,072	85.5	2,209,055	53.1	3,615,165	86.9	3,076,167	74.0

Data from the French national Covid vaccination (VACSI) database: https://geodes.santepubliquefrance.fr.
